# Gamma-Ray Sensor Using YAlO_3_(Ce) Single Crystal and CNT/PEEK with High Sensitivity and Stability under Harsh Underwater Conditions

**DOI:** 10.3390/s21051606

**Published:** 2021-02-25

**Authors:** Chanki Lee, Hee Reyoung Kim

**Affiliations:** Department of Nuclear Engineering, Ulsan National Institute of Science and Technology, 50 UNIST-gil, Ulju-gun, Ulsan 44919, Korea; lck1992@unist.ac.kr

**Keywords:** gamma-ray spectrometry, YAlO_3_(Ce), CNT/PEEK, underwater conditions, gross efficiency, photopeak efficiency

## Abstract

A new gamma-ray sensor, which could be employed in harsh underwater conditions, was developed using YAlO_3_(Ce) single crystal and carbon nanotube reinforced polyetheretherketone (CNT/PEEK). The sensor is compact, highly sensitive and stable, by providing real-time gross counts and an accumulated spectrum for fresh, saline, or contaminated water conditions. The sensor was tested in a water tank for quantification of the limit of detections. The Φ51 × 51 mm^2^ YAlO_3_(Ce) crystal exhibits a nearly perfect proportionality with a correlation of over 0.999 in terms of light yield per energy and possesses a high energy resolution. The chemically stable CNT/PEEK window material further enhances the detection efficiency by minimizing the background counts from penetrating gamma-rays. Data timeliness was obtained for regulation-based minimum detectable activity targets within 300 s. For a source-detector distance of up to 300 mm in water, the gross counts demonstrate the existence of radionuclides (Cs-137 and Co-60), owing to their higher efficiency (max. ~15 times) than those of the photopeak counts. Such differences between efficiency values are more likely in water than in air because of the high density of water, resulting in an increased build-up of scattered photons. The proposed sensor is suitable for autonomous underwater systems.

## 1. Introduction

Analyzing the radioactivity in natural water and in potentially nuclear-contaminated water is crucial for ensuring public safety and security. The environmental radioactivity of fresh surface water and saline seawater has been surveyed in countries and the results have been published periodically [1]. In addition, the radiological assessment and surveillance of spent nuclear fuel storage pools have been conducted considering the aspects of nuclear safety to avoid the release of radioactive materials into the environment during accidents or decommissioning of nuclear power plants [2].

To obtain timely radiation data for emergency responses, as in the case of the Fukushima Daiichi nuclear accident [3], on-site radionuclide identification techniques for underwater environments have been developed. Most techniques focused on gamma-ray spectrometry based on NaI(Tl) crystal as a high-energy gamma-ray wavelength shifter [4,5,6,7,8]. In addition to NaI(Tl) crystals, other materials such as LaBr_3_(Ce) [9], Gd_3_Al_2_Ga_3_O_12_(Ce) [10], and CeBr_3_ [11] have been tested to improve optical properties such as energy resolution, light yield, and/or stability.

Though several studies considered marine radioactivity measurements, the development of an autonomous sensor system is essential for effective and stable implementation under harsh underwater conditions. For instance, in 2019, we conceptually developed an autonomous underwater gamma radiation monitoring system that measured radioactivity at depths up to 2000 m, and relevant methods that were based on large volume YAlO_3_(Ce) single crystals and an autonomous underwater vehicle (AUV) were implemented [12].

In addition to the radiation sensor system, many environmental sensor systems have been studied by other researchers for autonomous underwater applications, improving design aspects such as stability, costs, compactness, handling ease, networking [13], path planning [14], or sensor accuracy itself. For example, Zhang et al. developed a new self-powered sensor by a piezoelectric diaphragm for detecting underwater disturbances, and the sensor performance was tested in water flows with different frequencies [15]. Renner et al. developed an open-source acoustic modem for AUVs underwater communication, being small enough to be on microAUVs, cost-effective, and having a low-power comsumption, covering long communication distances, and providing convenient ranging support [16]. Kang et al. conducted a stress and buckling analysis of a ray-type hull structure of a new underwater glider based on carbon fiber, being lighter and stronger than duralumin [17]. Wong et al. reported that operability under sea ice conditions, localization without GPS, vehicles based on multi thrusters, and intelligent manipulation skills have been the key technologies of autonomous robotics for deep-sea conditions [18]. 

Such multidisciplinary efforts imply that the future underwater radiation sensor should achieve at least compactness and durability, and be suitable to adopt new types of materials for better performance in harsh conditions. In this process, it is expected that sensors will naturally evolve into autonomous systems by incorporating other types of sensors, communication networks, intelligent handling, and data analysis.

Our previous study was limited in that it focused on theoretical analysis with assumptions, and the system design was not tested. Moreover, YAlO_3_(Ce) crystals had been regarded until then as a scintillation material for X-rays with lower energies than gamma-rays. Therefore, in this study, we developed a practical gamma-ray sensor using new types of materials like a Φ51 × 51 mm^2^ sized YAlO_3_(Ce) crystal and carbon nanotube reinforced polyetheretherketone (CNT/PEEK) composites, based on the design objective given in Section 2. We evaluated the sensor performance using a water tank, which is described in Section 3. We assessed the quantitative performance by analyzing the proportionality, energy resolution, detection efficiency, and background counts of the sensor. The applicability of the sensor design in the surface contamination scenario is discussed in Section 4, while cost issues were not taken into account in this study.

## 2. Performance-Oriented Sensor Design

### 2.1. Design Requirements

To optimize the sensor design, appropriate sensing components such as scintillation material, housing material, power sources, and communication modules were selected and combined, based on the performance of each component under specific underwater conditions. Quantitative requirements for the criteria are listed in Table 1. We aimed at fabricating a sensor that is sufficiently compact to be deployed in commercial remotely operated vehicles (ROVs), while satisfying the regulation-based minimum detectable activity (MDA) target of each gamma radionuclide within 300 s for every operating condition. The MDA target for gamma radionuclides was set as 40 kBq/m^2^ for surface contamination [19]. The operating time, temperature, salinity, pH, and pressure were fixed to ensure sensor stability under versatile underwater conditions such as freshwater, seawater, and spent nuclear fuel pool.

In addition to the quantitative design objective, we verified the ability of the sensor to form an autonomous system by coordinating with other required components, which were derived from the previous study.

### 2.2. Sensing Materials 

As mentioned in Section 1, we considered YAlO_3_(Ce) as a promising scintillation material [12,20], along with Gd_3_Al_2_Ga_3_O_12_(Ce), owing to the desirable features of non-hygroscopicity, mechanical hardness (none of the cleavage planes, scratch hardness ≥8.5 Mohs), short decay time (≤150 ns), and fair photoelectron yield and well-coordinated wavelength with the conventional photomultiplier tubes (PMTs). In particular, their relatively high radiation hardness is advantageous for precise and accurate gamma-ray measurements without any degradation under high background radiation conditions such as in medical and space applications [21,22]. Therefore, we considered both types of crystals as candidates for the gamma-ray sensing materials, and their performances were compared with those of the conventional NaI(Tl) scintillator of identical sizes. Table 2 indicates the major characteristics, where NaI(Tl) has the lowest mechanical and chemical stability among the three types of crystals, despite its highest production rate of photoelectrons. It was found that YAlO_3_(Ce) crystals are more effective than Gd_3_Al_2_Ga_3_O_12_(Ce) crystals, in terms of neutron insensitivity and photoelectrons/photons ratio.

To ensure the sensor compactness and its effectiveness in providing timely data, a computational simulation was conducted using the Monte Carlo N-Particles 6 code [23], considering the chemical compositions and densities of the crystals. Based on the simulation, where an 8% energy resolution at 662 keV (photopeak of Cs-137, one of the major gamma contaminants of interest) was assumed by the Gaussian energy broadening card, the optimal crystal size was determined to be Φ51 × 51 mm^2^. Accordingly, the abovementioned crystals were investigated with the derived size [Scionix and Epic Crystal] to test their energy resolutions. In optical coupling with PMTs of the same diameter, the obtained energy resolutions of YAlO_3_(Ce) and Gd_3_Al_2_Ga_3_O_12_(Ce) at 662 keV were 6.3% and 7.6%, respectively. YAlO_3_(Ce) exhibited a higher energy resolution than NaI(Tl), which was 6.9%. Such high energy resolution is theoretically correlated with the high proportionality in terms of the light yield for energy [20,24], which is a significant factor in stability. Additionally, high energy resolution improves the photopeak identification capability of gamma radionuclides and signal-to-background ratios, while an effective proportionality affects the accuracy and precision in the measurement of radioactivity. This tendency increases with the increase in crystal volume. Considering the overall features of YAlO_3_ (Ce), it was considered as a more suitable material than Gd_3_Al_2_Ga_3_O_12_(Ce) after comparing their features in Table 2, showing that it has effective mechanical stability and sensing performance.

**Figure 1:**
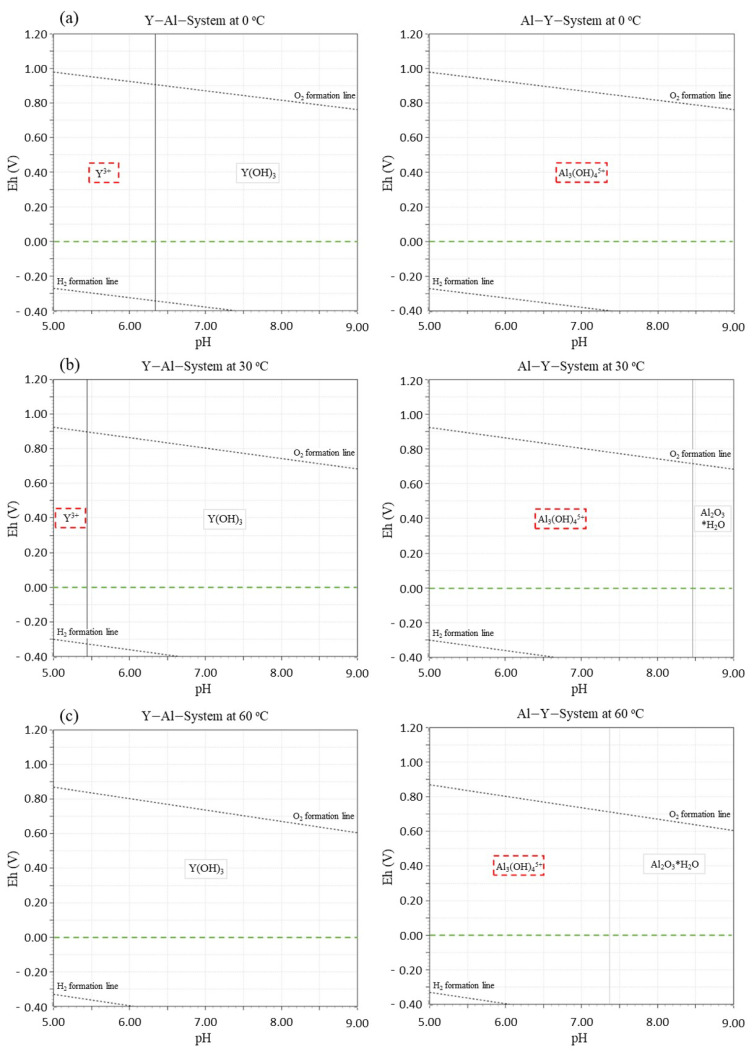
Eh-pH diagrams of YAlO_3_ for Y (**left**) and Al (**right**) at (**a**) 0 °C, (**b**) 30 °C, and (**c**) 60 °C in fresh water.

**Figure 2:**
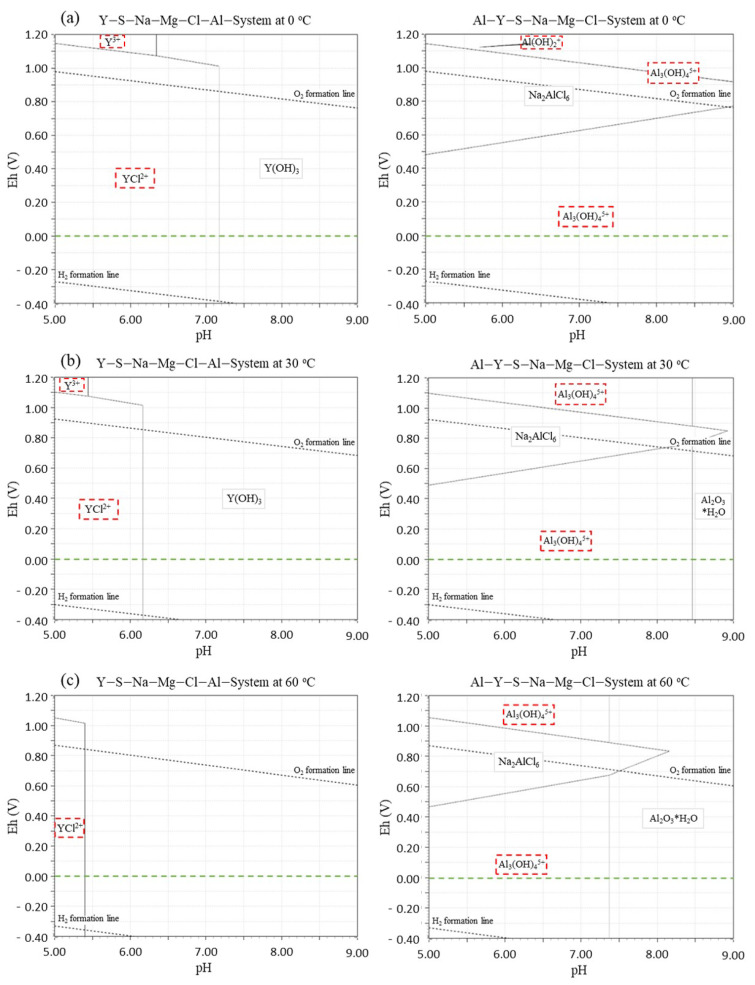
Eh-pH diagrams of YAlO_3_ for Y (**left**) and Al (**right**) at (**a**) 0 °C, (**b**) 30 °C, and (**c**) 60 °C in saline water.

To evaluate the thermodynamic stability of YAlO_3_(Ce) for the pH and temperature ranges given in Section 2.1, Eh-pH diagrams were plotted in fresh and saline water using the HSC chemistry code [25]. Using the diagrams, the corrosion resistance of the sensor materials in water was electrochemically assessed in the case of direct contact because of a leak through the broken housing set-up. Specifically, in the case of saline water, Cl, Na, Mg, and S concentrations were assumed to be 0.55 M, 0.47 M, 0.054 M, and 0.085 M, respectively, based on the real seawater compositions. Eh potential values of interests were set between −0.4 V to 1.2 V. As shown in Figure 1 and Figure 2, despite the non-hygroscopicity, unwanted chemical reactions that degrade the crystals occur by forming soluble phases such as Y^3+^, YCl^2+^, Al_3_(OH)_4_^5+^, or Al(OH)_2_^+^, especially in low pH and low-temperature saline water. Although the probability of encountering such conditions is low (seawater typically has a pH higher than 8.0 and the spent fuel pool temperature is not low as 0 °C), multiple barriers were adopted outside YAlO_3_(Ce) to reduce the possibility of direct water contact to zero. Specifically, the YAlO_3_(Ce) single crystal was surrounded by a reflector and Al body with a thickness of 1.5 mm and was coupled with a short PMT with 77 mm length and with the same diameter [R10131-01, Hamamatsu Photonics] for compactness.

The specifications of the PMT, as shown in Table 3, indicate that it matched well to the YAlO_3_(Ce) in terms of size, optical efficiency, time response, etc. The operating temperature was considered the only criterium that failed, but it was thought that heat transfer from the high temperature outside could be lowered by the air layer between the PMT and the housing.

### 2.3. Housing Materials

Watertight housing protects the sensing material, battery, and relevant circuits from corrosive, hot, and pressurized water. Al-6061 alloy was selected as the housing material for the body and back endcap, and the optimal alloy thickness of each side (t_body_, t_cap_) was calculated as 3 mm using Equations (1) and (2), respectively [26], under a safety factor of 3.

(1) $$t_{\mathrm{body}}=\frac{P\cdot R}{3S-0.6P}$$

(2) $$t_{\mathrm{cap}}=\frac{P\cdot \left(R+t_{\mathrm{body}}\right)}{3S}$$

where:

P is the pressure (MPa);

R is the inside radius (mm);

S is the maximum allowable stress (MPa).

The housing was then coated with 80 μm-thick Teflon to provide multiple barriers from water and light intrusion. While Al-6061 alloy has been reported as an efficient pressurizing material [27], a different material was chosen for the front endcap, which acted as a window for gamma-ray penetration. Generally, Be is used as the window material because of its low effective atomic number (4) and low density (1.85 g/cm^3^) [28]. However, unlike in pure water, Be dissolves in acids and base solutions, and is sensitive to impurities such as Cl, S, and F contained in water [29], thus the usage of Be is inappropriate in boric acid and seawater with the pH ranges of 5.0–7.0 and approximately 8.0, respectively.

Instead, CNT/PEEK was selected because of its compatibility with most of the chemicals and other advantages such as its black color that absorbs lights, high tensile strength (115 MPa), low effective atomic number (~6.3 [30]), and low density (1.31 g/cm^3^). Additionally, by adding CNT to pure PEEK, the electrical conductivity increases rapidly from ~$10^{-15}$ S/cm to ~$10^{-5}$ S/cm [31], thus reducing static electricity, which one should be aware of during radiation measurements. Both side endcaps and body enclosure were sealed together with screws and O-rings.

### 2.4. Power and Communication

**Figure 3:**
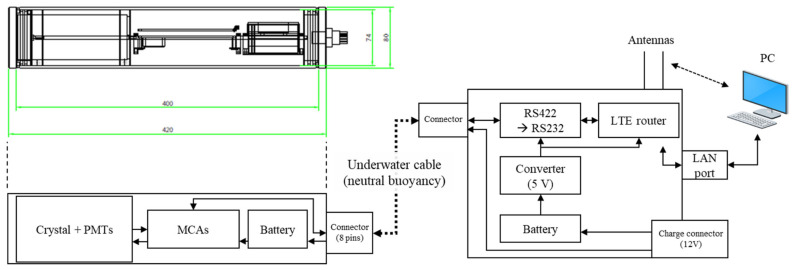
Schematic of the gamma-ray sensor.

Power source, communication modules, sensing material, and PMT were contained inside the housing with a dimension of Φ80 × 420 mm^2^ (Figure 3). A customized multichannel analyzer (MCA) circuit was used to ensure compactness with two laminated layers of analog/digital printed circuit boards. The MCA processed the PMT signals and stored the data in a microcontroller unit. As shown in Table 1, the data processed in the MCA included a gross count for every 1 s and the integrated gamma-ray spectrum from 1024 energy channels for 300 s. The data were transmitted onto the surface by RS-422 communication through eight pins underwater cable with neutral buoyancy (7GAX4 cable and MCBH-8 connector, SubConn Inc., Esbjerg, Denmark). The remaining cable pins, other than the two pairs (4 pins) used for communication, were also capable of simultaneously charging the built-in Li ion battery pack for the PMT and the remotely controlled MCA (on/off) using a toggle switch. Once the underwater data were sent to the surface, the communication module on the surface transmitted the overall data to an end-user PC through an LTE router. When using the 7.4-V 10400-mAh battery pack, the sensor current and the relevant expected lifetime in absence of the external charger were measured as 220 mA and ~47 h, respectively.

## 3. Sensor Testing

The gamma-ray sensor was tested in the Korea Laboratory Accreditation Scheme (KOLAS) quality assurance system, which includes saline water, temperature and humidity, vibration, thermal shock, and mechanical shock tests. KOLAS successfully demonstrated that the sensor was stable in the given temperature range and was water-resistant.

**Figure 4:**
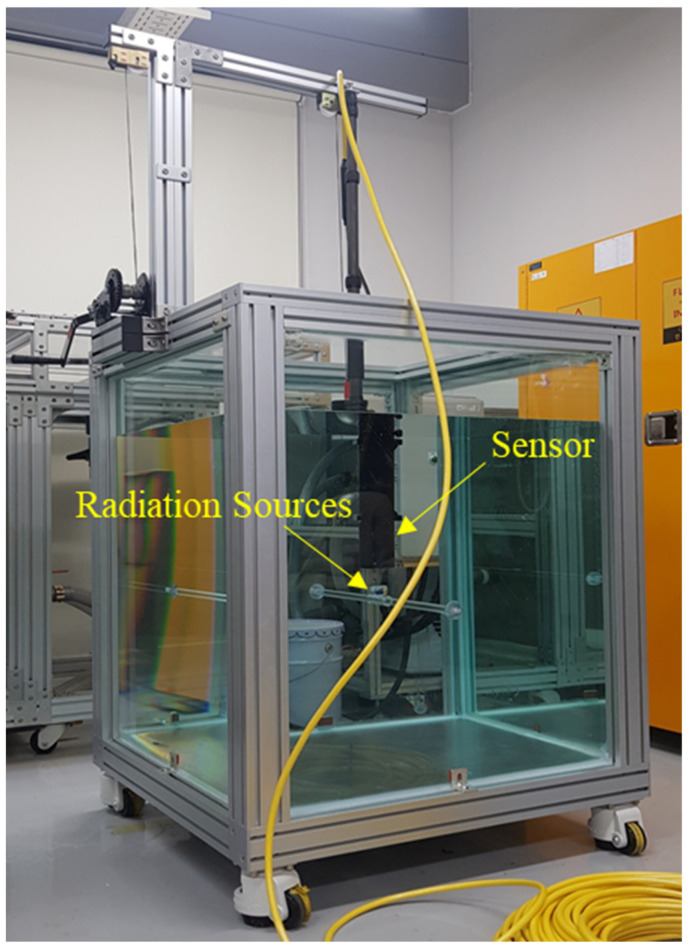
Water tank used for sensor calibration.

The sensing stability and sensitivity were also analyzed by measuring the sensing performance. Specifically, the sensor was calibrated by the measurement of gamma-ray sources with a known radioactivity concentration in a water tank with dimensions of 0.8 × 0.8 × 1.0 m^3^ (shown in Figure 4). In this study, fresh tap water with low background radioactivity was filled in the tank, assuming a simple surface contamination scenario prior to volumetric diffusion of contaminants.

Proportionality and energy resolution data were calibrated using photopeaks from artificial radionuclides (Am-241, Cs-137, and Co-60) and natural radionuclides (K-40, and Tl-208) to analyze the sensor stability. Here, the optimal supply voltage of the PMT to cover the energy up to 3 MeV was fixed at 700 V. Subsequently, the gross and photopeak counts from Cs-137 and Co-60 sealed sources were obtained via a spectrometer for 1800 s. The results, which included various gamma-ray effective ranges, detection efficiency, and MDAs, were quantitatively analyzed to assess the usefulness of each data type, gross counts, and spectrum, to find radioactive contaminants with a different source-detector distances and measurement time.

The theoretical basis for the study was developed to discuss the parameters above. We first quantified the maximum effective range of gamma-rays (R_γ_) with a specific energy in a specific medium as given by Equation (3), which was defined based on the exponentially decaying nature of attenuation [12].

(3) $$R_{\gamma }=-\frac{\ln\left(P_{\mathrm{eff}}\right)}{\mu \left(E\right)\cdot \rho }$$

where

P_eff_ is the cut-off probability for gamma-ray attenuation;

µ(E) is the mass attenuation coefficient at energy E (cm^2^/g);

ρ is the density of water (g/cm^3^).

Gross efficiency ($\epsilon_{\mathrm{gross}}$) and photopeak efficiency ($\epsilon_{\mathrm{photopeak}}$), which are affected by R_γ_, for each nuclide were calculated using Equations (4) and (5), respectively.

(4) $$\epsilon_{\mathrm{gross}}=\frac{{\mathrm{CPS}}_{\mathrm{gross}}}{I_{\gamma }\cdot \mathrm{DPS}}$$

(5) $$\epsilon_{\mathrm{photopeak}}=\frac{{\mathrm{CPS}}_{\mathrm{photopeak}}}{I_{\gamma }\cdot \mathrm{DPS}}$$

where

CPS_gross_ is the gross count per second;

CPS_photopeak_ is the photopeak count per second within ±1.5 full width at a half maximum (FWHM);

I_γ_ is the gamma-ray emission probability;

DPS is the disintegration per second for each nuclide;

For the estimation of efficiency values, specifically, background radiation data were obtained at the same distance and time as for the contaminated conditions but with the usage of dummy sources instead of radioactive sources. The baseline was then subtracted. While calculating the efficiency of Co-60 with two photopeaks (1173 keV and 1332 keV), the two efficiency values were averaged, and the uncertainty was propagated by assuming uncorrelated linear combinations. From the estimated efficiency, the Currie MDA with 95% confidence was calculated using Equation (6) [28].

(6) $${\mathrm{MDA}}_{95\%}=\frac{\left(2.71+4.65\sqrt{\mathrm{bkg}\cdot t}\right)}{\epsilon \cdot t}$$

where

bkg is the background count per second;

t is the measurement time (s);

ϵ is the gross or photopeak efficiency;

## 4. Results and Discussion

### 4.1. Proportionality and Energy Resolution

**Figure 5:**
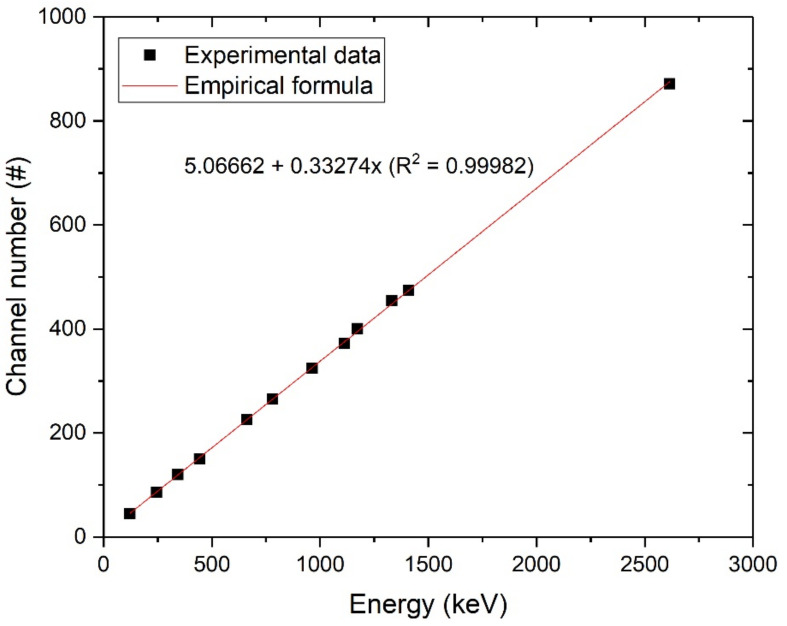
Linearity of channel numbers and energies.

**Figure 6:**
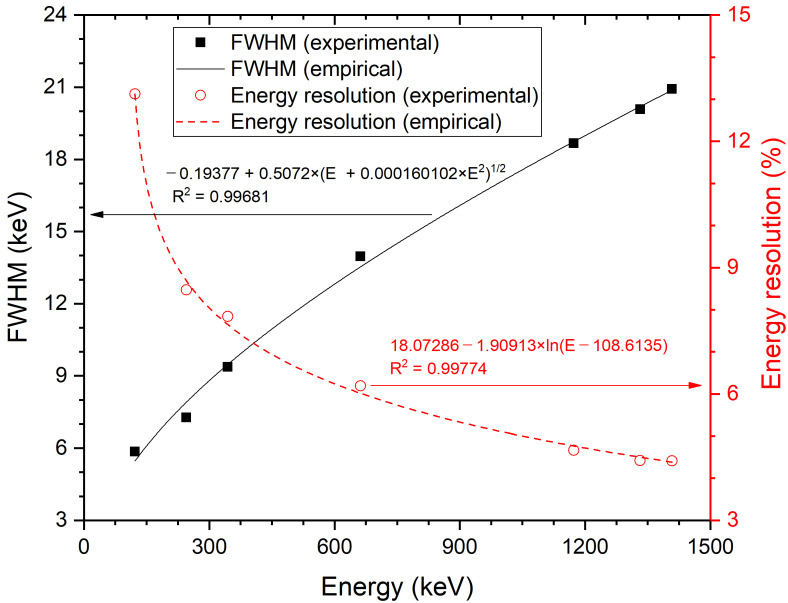
FWHM and energy resolution as functions of energy (in keV).

As shown in Figure 5, the linearity of counts for energies <3000 keV was well obtained from a single spectrum of mixed sources, where each peak channel and relevant FWHM were evaluated by Gaussian fitting. This verified the excellent proportionality of the YAlO_3_(Ce) crystal in terms of light yield, while more detailed analyses with different temperatures, applied voltages, or other varying conditions would be needed in the future. The statistical R^2^ value for the plot was >0.999. Because of the exceptional proportionality, the energy resolution of YAlO_3_(Ce) also followed the theoretical tendency (exponential and logarithmic correlation of R^2^ > 0.99) as shown in Figure 6.

### 4.2. Detection Efficiency

**Figure 7:**
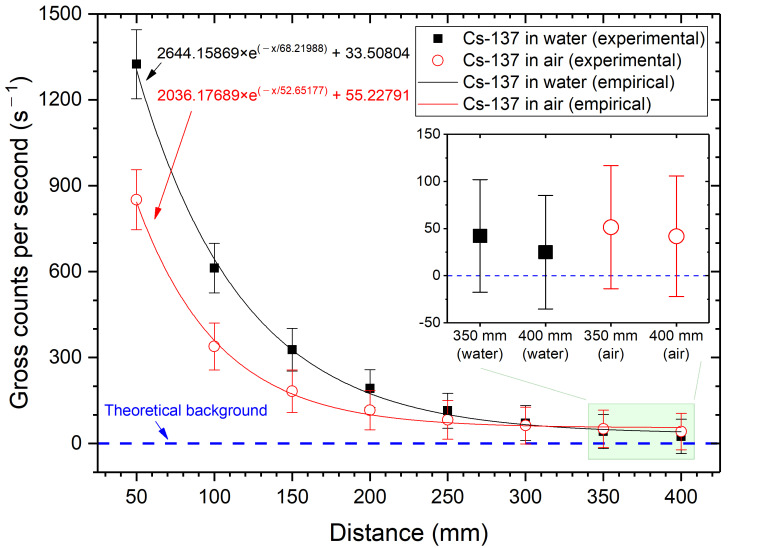
Gross counts per second of Cs-137 as a function of the distance.

**Figure 8:**
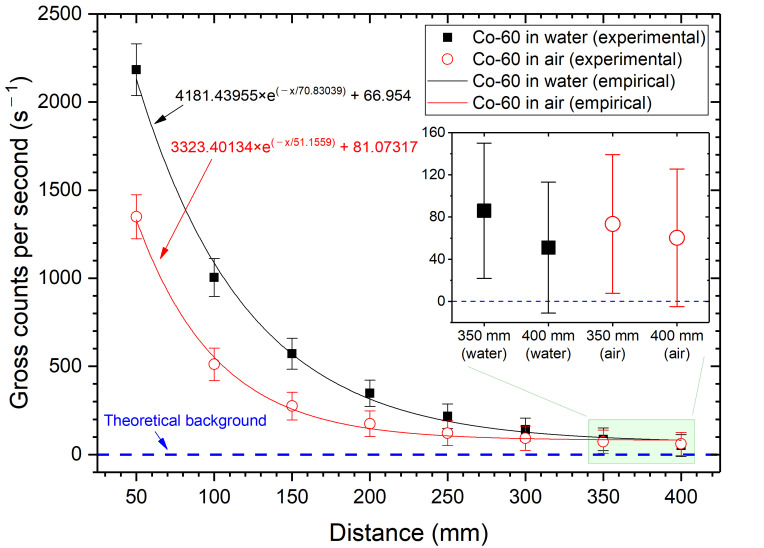
Gross counts per second of Co-60 as a function of the distance.

As shown in Figure 7 and Figure 8, Cs-137 and Co-60 indicate that the maximum effective range must be between 350 mm and 400 mm, considering 3-sigma standard deviations. The cut-off probability (P_eff_) value of 0.03, as in a previous study [12], was a reasonable assumption. However, values between 0.05 to 0.07 are preferable, based on the attenuation coefficient data from NIST XCOM. Nonetheless, we could not quantify a fixed probability value, and the statistically meaningful effective range was within 400 mm.

**Figure 9:**
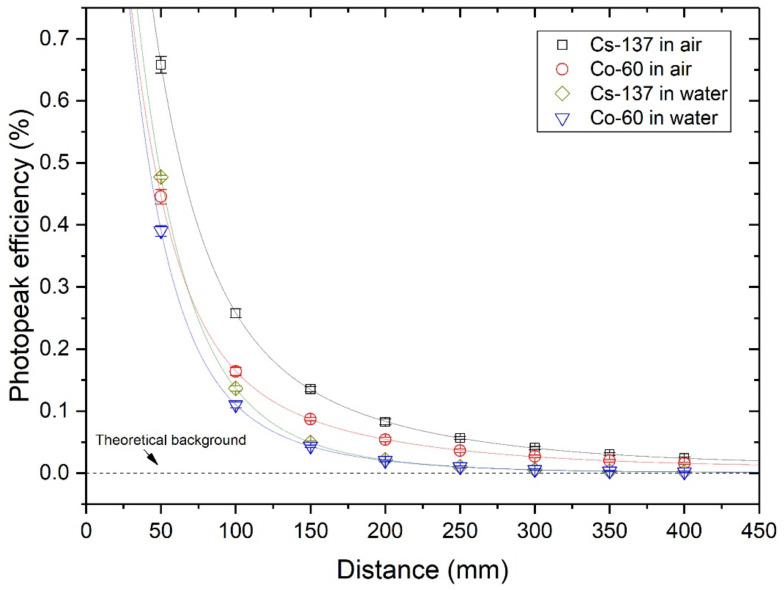
Photopeak efficiency for sealed sources at different distances and media.

**Figure 10:**
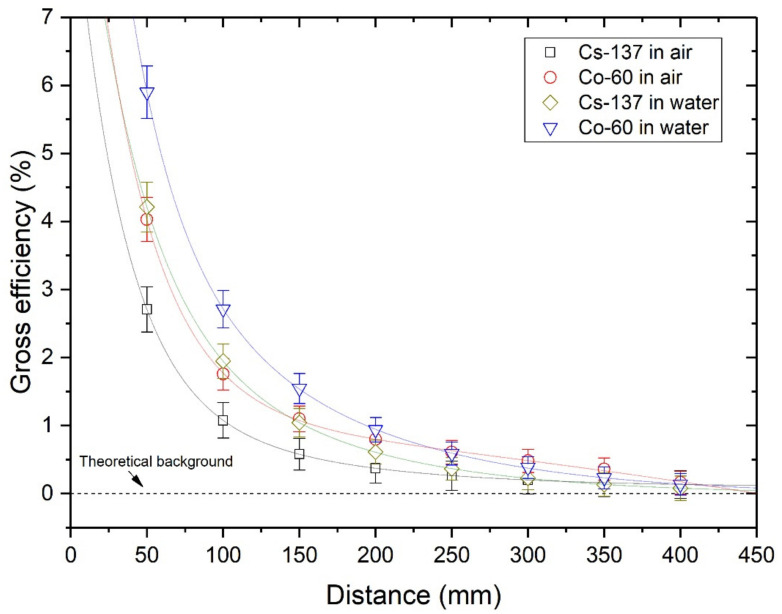
Gross efficiency for sealed sources at different distances and media.

The gross efficiency was higher than the photopeak efficiency under similar conditions as those shown in Figure 9 and Figure 10.

In particular, for Co-60 at 50 mm distance, the gross efficiency was ~15 times larger than the photopeak efficiency. In all cases, the photopeak efficiency was lower in water than in air. This was because of the different densities of these media, resulting in different probabilities of Compton scattering events. A high Compton scattering rate contributes to the reduction in photoelectric absorption rate and increases the build-up factor B(E,x), which is defined as the ratio of the total count rate to count rate only from the direct gamma-rays without scattering counts [28]. The relative build-up factor of water to air media can be approximately calculated as Equation (7), if the count rate in air is assumed to be the pure emission rate, I_0_.

(7) $$B\left(E,x\right)=\frac{I\left(E,x\right)}{I_{0}e^{-\mu \left(E\right)\rho x}}≅\frac{\epsilon_{\mathrm{water}}}{\epsilon_{\mathrm{air}}e^{-\mu \left(E\right)\rho x}}$$

where

x is the source-detector distance (cm);

$\epsilon_{\mathrm{water}}$ is the gross or photopeak efficiency in water;

$\epsilon_{\mathrm{air}}$ is the gross or photopeak efficiency in air.

By calculating the relative build-up factors as well as their ratios for gross counts to those for photopeak counts, we indirectly quantified the build-up contributions, as presented in Table 4 and Table 5, respectively. For these analyses using mixed data, one more decimal place was set for the uncertainty as compared to the average values [32]. Obviously, both ratios increase as distance increases. While ratios of total counts to photopeak counts were larger for Cs-137 than Co-60, their ratios in water to those in air were larger for Co-60 than Cs-137.

**Figure 11:**
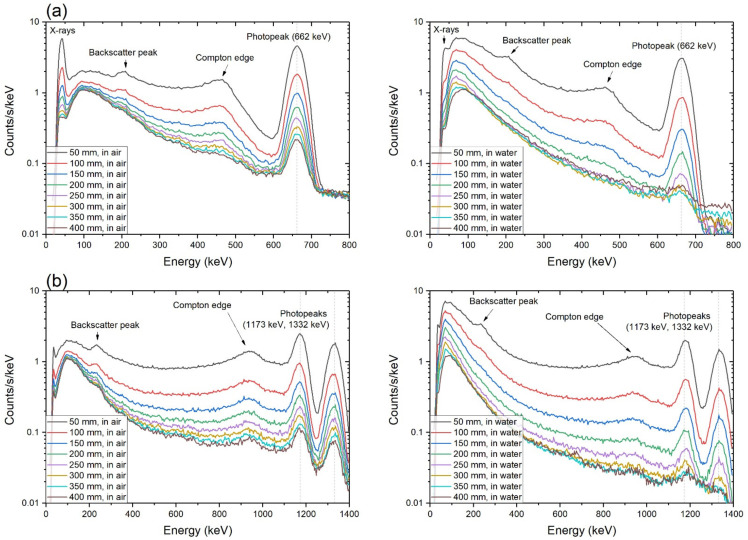
Gamma-ray spectra of (**a**) Cs-137 and (**b**) Co-60 obtained at 50–400 mm distances in air and in water for 300 s.

Such build-up contributions can be observed in the energy spectra (Figure 11). X-rays, backscatter peaks, Compton edges, and photopeaks, which are prominent in air, fade in water as the distance increases. Additionally, the built-up counts are concentrated on a low-energy region smaller than ~250 keV. From the shape of the spectra in a lower energy region compared to the photopeak, it is confirmed that CNT/PEEK window nearly not interrupts the incident gamma-ray from penetrating into the scintillator crystal.

**Figure 12:**
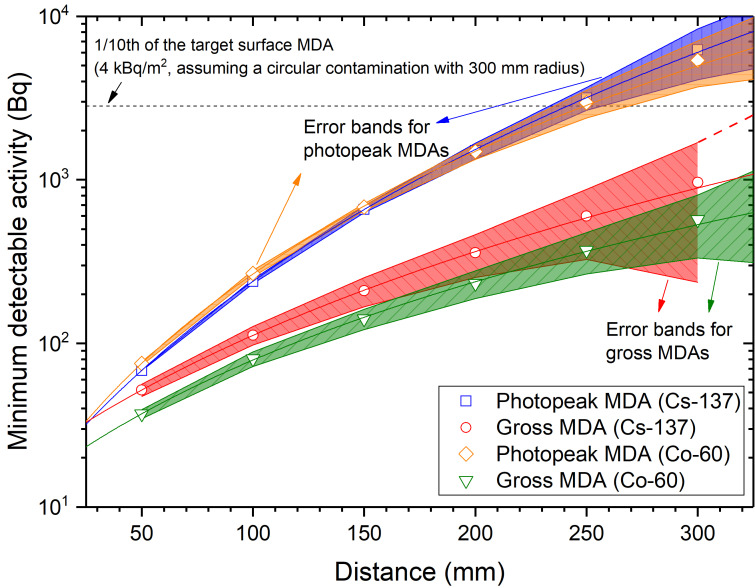
Minimum detectable activity for the photopeak and gross minimum detectable activity (MDA).

As shown in Figure 12, the gross counts can provide more efficient information for the rapid localization of contaminations in water than in air, where the differences in densities and attenuation coefficients of air and water cause changes in the build-up factor from Compton scattering, thereby altering the gross efficiencies and relevant MDAs for 300 s measurements. In all cases with distances less than ~250 mm, the achieved surface contamination MDAs for 300 s, assuming circular contamination with 300-mm radius for a check source, were even ten times smaller than our MDA target, 40 kBq/m^2^. The power of gross counts will be higher in actual surface contamination than in our experimental condition, because we placed the source away from the water tank bottom side (~200 mm), thereby underestimating the build-up contribution from backscattering with an angle of ~180°.

**Figure 13:**
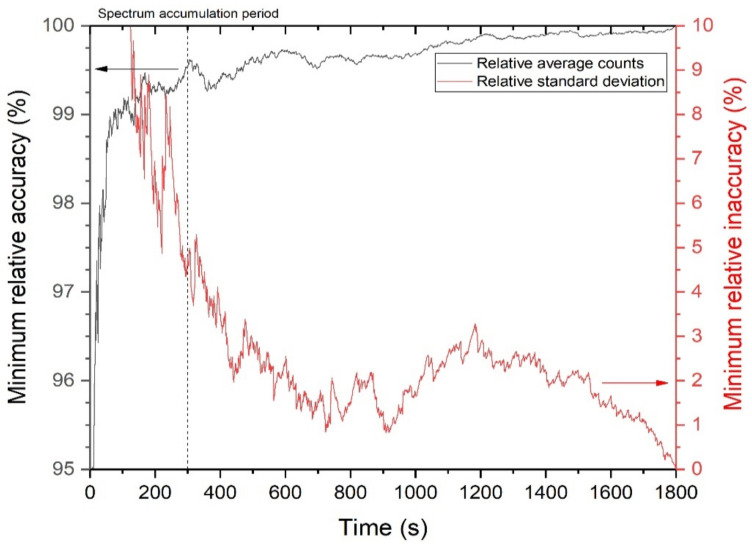
Minimum accuracy and maximum inaccuracy (in %) as a function of time.

Figure 13 shows the minimum relative accuracy and maximum relative inaccuracy of average counts and standard deviation, respectively. We defined these indicators as conservative measures of how close and far a value at a certain time is among the dataset measured repeatedly, to a reference value in percent relative range. In particular, we utilized six datasets and determined the reference values as those at 1800 s. As a result, the gross count data accumulated for a measurement time of 300 s are sufficiently converging to the reference values, with ~99% accuracy. The standard deviation for 300 s was comparable to the reference values, with an inaccuracy of only ~5%. Thus, the spectrum accumulation period of 300 s was sufficient to assure the data quality, while satisfying our target MDA. Even so, it should be noted that secondary peaks, which lower the quality, may occur after 300 s due to statistical fluctuations, as reflected in the inaccuracy value at ~1200 s of Figure 13.

## 5. Conclusions

In this study, we developed a new gamma-ray sensor based on the YAlO_3_(Ce) single crystal and CNT/PEEK with the performance-oriented design objective of operation under harsh underwater conditions. Assuming surface contamination in fresh water, the YAlO_3_(Ce) crystal exhibited its capability to identify and quantify gamma radioisotopes of a 4 kBq/m^2^ MDA target (10 times lower than our design objective) within 300 s, using the gross counts and gamma-ray spectrum data. The CNT/PEEK in the sensor ensured high sensitivity and stability, and significantly reduced the attenuation degree of the incident radiation. While the sensor was not tested in various contamination conditions, we will test the volumetric contamination in the future, using open radiation sources both in fresh and saline water. In particular, the signal-to-background ratio will be calculated for different underwater conditions on the pilot-scale, which controls the temperature, pressure, salinity, and water-flow velocity. Ultimately, the sensor will be deployed in ROVs equipped with Bayesian estimation algorithms for autonomy.

## Figures and Tables

**Table 1:** Design objective of the underwater gamma-ray sensor.

Criteria	Requirements
mass and size	ROV-deployable and hand-held size (<3 kg)
data timeliness	gamma-ray spectrum for every 300 s gross counts for every 1 s
MDA target (nuclide)	40 kBq/m^2^ (gross, surface contamination)
energy range	up to 3 MeV (1024 energy channels)
operating time	at least 24 h without charging
operating temperature	0–60 °C
operating salinity	up to 3.5 wt%
operating pH (lifespan)	5.0–9.0 (>1 yr)
operating pressure	up to 20 bar (~200 m depth)

**Table 2:** Major characteristics of YAlO_3_(Ce), Gd_3_Al_2_Ga_3_O_12_(Ce), and NaI(Tl).

	YAlO_3_(Ce)	Gd_3_Al_2_Ga_3_O_12_(Ce)	NaI(Tl)
emission maximum (nm)	370	520	410
density (g/cm^3^)	5.37	6.63	3.67
hygroscopicity	no	no	yes
cleavage plane (Miller index)	none	none	<100>
scratch hardness (Mohs)	8.5	8	2
radiation hardness (Gy)	$10^{4}$	$10^{3}$	10
neutron sensitivity	no	yes	yes
decay time (ns)	25–30	50–150	230
light yield (photons/keV)	25	50	40
photoelectron yield (phe/keV)	3.5–4	6.2	10
ratio of photoelectron/light (phe/photons)	0.14–0.16	0.124	0.25

**Table 3:** Specifications of the used photomultiplier tube (PMT) [R10131-01, Hamamatsu Photonics].

Specification Parameter (unit)	Value
operating temperature (°C)	−30 to +50
spectral response (nm)	300 to 650
quantum efficiency (%, at 390 nm and at 25 °C)	30
max. supply voltage (V)	1500
voltage gain	2.7 × $10^{5}$
dark current (nA)	2–20
rise time (ns)	7.3
transit time (ns)	49
pulse linearity (mA, ±2% deviation)	1

**Table 4:** Relative build-up factors in water to in air.

	Cs-137, Gross	Cs-137, Photopeak	Co-60, Gross	Co-60, Photopeak
5 mm	2.39 ± 0.360	1.11 ± 0.023	2.01 ± 0.208	1.20 ± 0.031
10 mm	4.27 ± 1.170	1.25 ± 0.036	2.91 ± 0.482	1.26 ± 0.051
15 mm	6.53 ± 2.945	1.33 ± 0.047	3.65 ± 0.816	1.29 ± 0.044
20 mm	9.20 ± 6.041	1.46 ± 0.099	4.19 ± 1.224	1.31 ± 0.103
25 mm	11.86 ± 11.056	1.56 ± 0.106	4.71 ± 1.879	1.36 ± 0.142
30 mm	14.99 ± 19.075	1.71 ± 0.220	5.33 ± 2.905	1.40 ± 0.153
35 mm	16.67 ± 30.138	2.04 ± 0.359	5.93 ± 4.898	1.50 ± 0.267
40 mm	18.66 ± 50.839	2.67 ± 1.027	10.70 ± 16.419	1.55 ± 0.426

**Table 5:** Ratios of relative build-up factors for gross counts to those for photopeak counts.

	Cs-137	Co-60
5 mm	2.15 ± 0.326	1.68 ± 0.178
10 mm	3.41 ± 0.940	2.31 ± 0.394
15 mm	4.92 ± 2.229	2.83 ± 0.641
20 mm	6.28 ± 4.149	3.21 ± 0.970
25 mm	7.58 ± 7.084	3.46 ± 1.426
30 mm	8.77 ± 11.220	3.82 ± 2.123
35 mm	8.16 ± 14.829	3.95 ± 3.337
40 mm	7.00 ± 19.263	6.92 ± 10.794
